# Response to letter to the editor entitled “Association between left atrial sphericity index and clinical outcomes in patients with systolic heart failure”

**DOI:** 10.1002/clc.23563

**Published:** 2021-02-08

**Authors:** Mayu Yazaki, Takeru Nabeta, Junya Ako

**Affiliations:** ^1^ Department of Cardiovascular Medicine Kitasato University School of Medicine Sagamihara Japan

To the Editor

We thank Dr. Chapman and Dr. Imamura for their letter regarding our article. We agree with the point that left atrial (LA) size affects the hemodynamic status. In our study, cardiac magnetic resonance (CMR) examinations underwent at discharge (n = 159, 62.4%) and stable ambulatory settings (n = 96, 37.6%). CMR test requires a long inspection time and breath‐holding, therefore, it cannot be performed in patients with unstable hemodynamic condition. We agree that it is meaningful whether the LA sphericity index is a changeable parameter with heart failure therapy. Decreasing LA volume is associated with improvement in left ventricular contractility,^1^ thus it is assumed that the LA sphericity index also may change with medical therapy such as diuretics. We did not measure the follow‐up data of the LA sphericity index. For this reason, it is unclear how much the LA sphericity will change with heart failure therapy. The time course of the LA sphericity index is an important theme, therefore future research will be needed.

It is preferable to perform cardiac catheterization simultaneously as CMR examinations to obtain detailed pressure and histological data. However, this study was a retrospective study, so it was difficult to survey them. For the same reason, we have not been able to measure endocrine or inflammatory makers. As you indicated an elevated inflammatory response, such as C‐reactive protein, interleukins, and cytokines, was shown to be involved in the geometric change of LA.^2^ Further prospective research will need to reveal the relationship among endocrine parameters, inflammatory markers, and the LA sphericity index.

To identify the cutoff value of the LA sphericity index for a cardiac event, we performed receiver operating characteristic (ROC) analysis. The ROC analysis revealed that the cutoff value of the LA sphericity index of hospitalization for heart failure was 0.78 (p < .001, Figure 1). However, we did not include it in the manuscript because we believed that it can be important to note that an elevated the LA sphericity index is associated with heart failure hospitalization rather than focusing on a specific cutoff value in the clinical practice.

**FIGURE 1:**
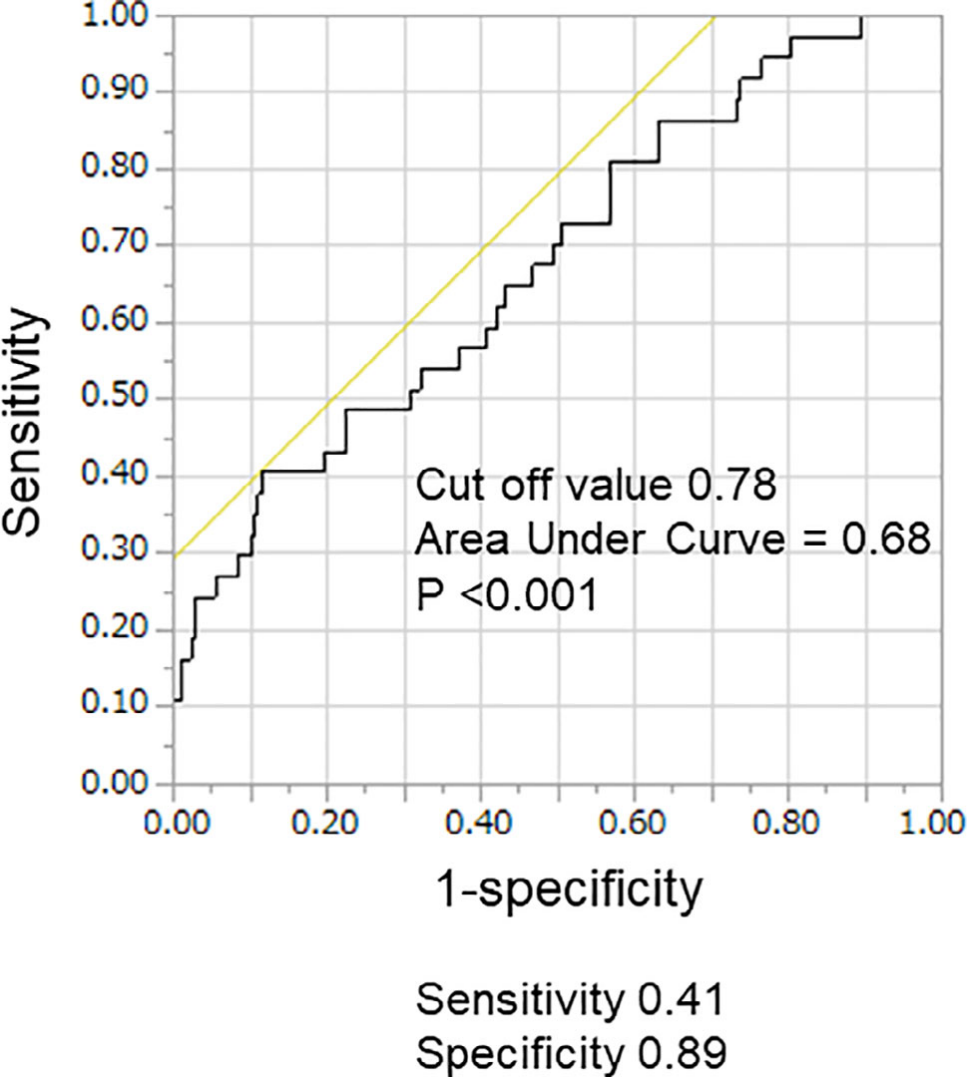
Receiver operating characteristic curve for left atrial sphericity index

## CONFLICT OF INTEREST

None.
